# Altered Metabolic Flexibility in Inherited Metabolic Diseases of Mitochondrial Fatty Acid Metabolism

**DOI:** 10.3390/ijms22073799

**Published:** 2021-04-06

**Authors:** Sara Tucci, Khaled Ibrahim Alatibi, Zeinab Wehbe

**Affiliations:** 1 Department of General Pediatrics, Adolescent Medicine and Neonatology, Faculty of Medicine, Medical Center-University of Freiburg, 79106 Freiburg, Germany; Khaled.ibrahim.alatibi@uniklinik-freiburg.de (K.I.A.); Zeinab.wehbe@uniklinik-freiburg.de (Z.W.); 2 Faculty of Biology, University of Freiburg, Schaenzlestrasse 1, 79104 Freiburg, Germany; 3 Center of Pediatric and Adolescent Medicine-Medical Center, Department of Pediatric Hematology and Oncology, Faculty of Medicine, University of Freiburg, 79106 Freiburg, Germany

**Keywords:** metabolic flexibility, mitochondrial fatty acid metabolism, inherited metabolic disorders, mtFAS, VLCADD

## Abstract

In general, metabolic flexibility refers to an organism’s capacity to adapt to metabolic changes due to differing energy demands. The aim of this work is to summarize and discuss recent findings regarding variables that modulate energy regulation in two different pathways of mitochondrial fatty metabolism: β-oxidation and fatty acid biosynthesis. We focus specifically on two diseases: very long-chain acyl-CoA dehydrogenase deficiency (VLCADD) and malonyl-CoA synthetase deficiency (acyl-CoA synthetase family member 3 (ACSF3)) deficiency, which are both characterized by alterations in metabolic flexibility. On the one hand, in a mouse model of VLCAD-deficient (VLCAD^−/−^) mice, the white skeletal muscle undergoes metabolic and morphologic transdifferentiation towards glycolytic muscle fiber types via the up-regulation of mitochondrial fatty acid biosynthesis (mtFAS). On the other hand, in ACSF3-deficient patients, fibroblasts show impaired mitochondrial respiration, reduced lipoylation, and reduced glycolytic flux, which are compensated for by an increased β-oxidation rate and the use of anaplerotic amino acids to address the energy needs. Here, we discuss a possible co-regulation by mtFAS and β-oxidation in the maintenance of energy homeostasis.

## 1. Introduction

The term “metabolic flexibility” was first used in the context of helminths to describe the generation of chemical energy and key metabolites that provided them with the metabolic flexibility to respond and adapt to changes in their environment [1]. In humans, this term refers to the selection of fuel by the organism, in different situations, toward fulfilling its energy needs by matching fuel availability [2]. Over recent years, the recognition of the importance of metabolic flexibility has increased remarkably, and it has been described to be an essential adaptive process in health and disease [2,3,4,5,6,7,8,9]. Several inherited metabolic diseases negatively affect energy metabolism, and its dysregulation in fatty acid oxidation disorders have been described [10,11,12,13,14,15]. In this study, we focus on two diseases of mitochondrial fatty acid metabolism, i.e., very long-chain acyl-CoA dehydrogenase deficiency (VLCADD) and malonyl-CoA synthetase deficiency (acyl-CoA synthetase family member 3 (ACSF3) deficiency). We discuss how monogenic diseases themselves as well as other variables, such as sex and diet, affect metabolic flexibility, and how both pathways interact to maintain energy homeostasis.

## 2. Mitochondrial Metabolism of Fatty Acids

Mitochondria are the powerhouse of the cells, generating up to 90% of the energy within a cell in the form of adenosine triphosphate (ATP). There is a close connection between fatty acid metabolism and mitochondria [16], involving a considerable number of cellular processes that go well beyond mitochondrial fatty acid metabolism [16]. Fatty acids are essential for ATP and energy production [17], and are therefore highly relevant in the regulation of energy homeostasis. The processes of β-oxidation, linked to ATP production, and mitochondrial fatty acid biosynthesis (mtFAS) are both localized in the mitochondria. This last pathway, in particular, produces molecules that are used as cellular structural components for post-translational modifications of proteins and in signaling cascades [16].

### 2.1. Mitochondrial β-Oxidation

**Figure 1:**
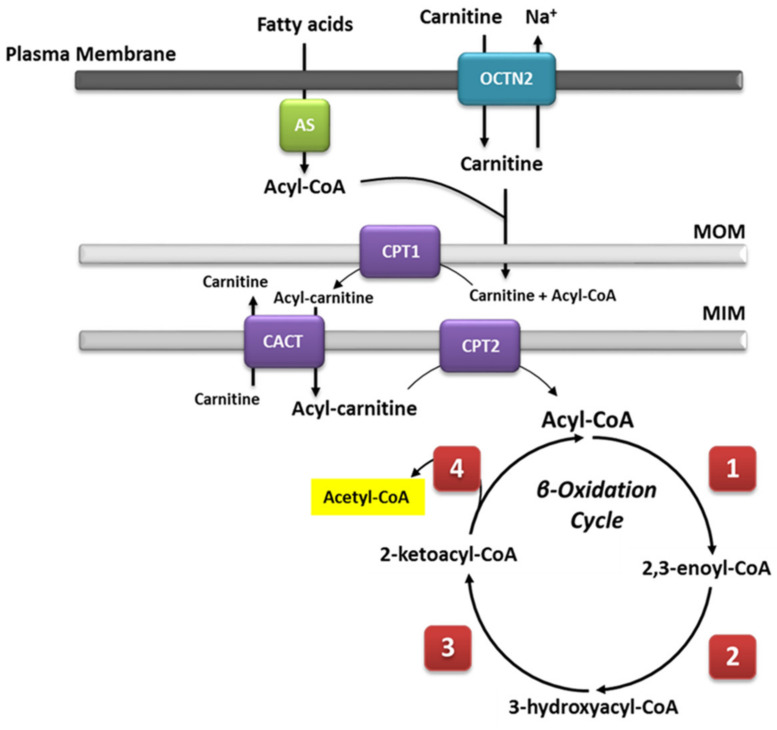
Schematic representation of the mitochondrial fatty acid β-oxidation. Long-chain fatty acids need to be transported into the mitochondria by an active transport system. Because fatty acids in the cytoplasm are activated as acyl-CoA esters, they must be bound to carnitine for transport across the mitochondrial membranes. In contrast, medium- and short-chain fatty acids do not need an active transport system and can easily undergo β-oxidation. This pathway consists of four cyclic steps. Each step shortens the acyl-CoA by two carbons. The electrons generated by each oxidative reaction are transferred to FAD or NAD+ and are forwarded to ubiquinone or the respiratory chain, respectively. (1) Acyl-CoA dehydrogenase (very long-, long-, medium-, and short-chain acyl-CoA dehydrogenase, i.e., VLCAD, long-chain acetyl-CoA dehydrogenase (LCAD), medium-chain acyl-CoA dehydrogenase (MCAD), and SCAD, respectively).; (2) 2,3-Enoyl-CoA hydratase (LCEH and SCEH). (3) 3-Hydroxyacyl-CoA dehydrogenases (LCHAD and SCHAD). (4) β-Ketoacyl-CoA thiolase (LCKAT, MCKAT, and SCKAT). MIM, mitochondrial inner membrane; MOM, mitochondrial outer membrane.

Mitochondrial β-oxidation is essential for energy production from fat. Short- and medium-chain fatty acids can cross the mitochondrial double membrane without the need for an active transport system and subsequently participate in the β-oxidation cycle [18]. By contrast, longer-chain fatty acids cannot passively cross the mitochondrial membrane [19]; for this purpose, the long-chain fatty acids are covalently bound by carnitine palmitoyltransferase I (CPT1) located on the mitochondrial outer membrane. Then, the synthetized acylcarnitines are transported across the membranes by carnitine-acylcarnitine translocase (CACT). Upon reaching the mitochondrial matrix, the acylcarnitines are then converted to active acyl-CoA and free carnitine by carnitine O-palmitoyltransferase 2 (CPT2), and they can then undergo β-oxidation. During this process, fatty acids in the form of acyl-CoAs are fully degraded to acetyl-CoA via cleavage of two carbons in each cycle of β-oxidation. The cleavage involves four steps that are dependent on four enzymes, i.e., FAD-dependent acyl-CoA dehydrogenase, enoyl-CoA hydratase, NAD-dependent L-β-hydroxyacyl-CoA dehydrogenase, and β-ketoacyl-CoA thiolase [20], each of which are characterized by specificity for fatty acids of different chain lengths [21]. A general overview is summarized in Figure 1. Saturated and unsaturated fatty acids can be fully degraded during β-oxidation; the only difference in the process is the need for additional enzyme isoforms, i.e., Δ3,Δ2-enoyl-CoA isomerase, Δ3,5,Δ2,4-dienoyl-CoA isomerase, and 2,4-dienoyl-CoA reductase, for the degradation of unsaturated fatty acids [22]. The acetyl-CoA unit, cleaved in each β-oxidation cycle, is used as substrate for the citric acid cycle (TCA), generating the required reduction equivalents (NADH and FADH2) for ATP synthesis during oxidative phosphorylation.

### 2.2. Very Long-Chain Acyl-CoA Dehydrogenase Deficiency (VLCADD)

Deficiencies in one or more enzymes involved in the β-oxidation pathway belong to the group of diseases known as fatty acid oxidation disorders (FAOD) and may induce life-threatening situations, such as coma, and death [23]. Especially affected are organs with a high metabolic rate, such as the heart, liver, and skeletal muscle. Very long-chain acyl-CoA dehydrogenase deficiency (VLCADD, OMIM 609575) is considered to be the most common inherited disease of the mitochondrial β-oxidation of long-chain fatty acids (LCFA). VLCAD deficiency is an autosomal recessive disorder with an incidence of 1:50,000 to 1:100,000 in newborns [24]. The main mechanisms involved in the development of symptoms include the following: (I) severe energy deficiency due to deficient fatty acid oxidation with consequent impairment in ketone body biosynthesis and (II) the accumulation of toxic, long-chain acylcarnitines. Overall, fasting and other catabolic situations in response to illnesses or prolonged exercise may induce clinical symptoms and severe metabolic derangement [24]. To date, no drug therapy for the treatment of VLCADD is yet available. The recommended therapeutic approaches include avoidance of fasting, with frequent meals to prevent catabolic states, and replacement of long-chain triglycerides with medium-chain triglycerides (MCTs). Indeed, after hydrolysis, MCTs are oxidized by the enzyme medium-chain acyl-CoA dehydrogenase (MCAD), bypassing the bottleneck represented by VLCAD and, therefore, MCTs are fully metabolized to supply the organism with the required energy. Triheptanoin, a medium-chain triglyceride with three odd-chain heptanoic acids, has recently been demonstrated to reduce the number of hospitalizations of long-chain FAOD patients and to maintain cardiac performance [25,26,27]. Two mouse models for VLCADD have been generated, the long-chain acetyl-CoA dehydrogenase (LCAD) knockout mouse [28] and VLCAD^−/−^ [29,30]. In particular, the VLCAD^−/−^ mouse model by Exil et al. [30] has been extensively characterized in recent years [11,31,32,33,34,35,36,37,38,39,40,41,42].

### 2.3. Mitochondrial Fatty Acid Biosynthesis

**Figure 2:**
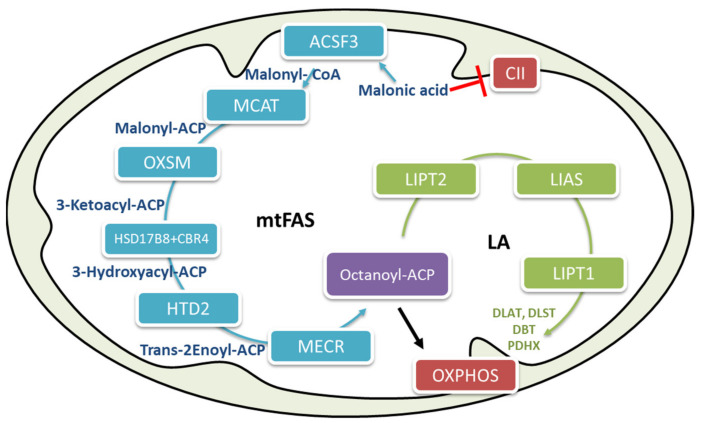
The human mitochondrial fatty acid biosynthesis (mtFAS) pathway. The mFAS pathway is shown in blue, and the lipoic acid biosynthesis pathway is shown in green. The end product of mFAS is octanoyl-ACP, which is used as a substrate for the biosynthesis of lipoic acid. Acyl-ACP actively participates in oxidative phosphorylation (OXPHOS) assembly. CII, complex II of the electron transport chain; LA, lipoic acid; ACSF3, acyl-CoA synthetase family member 3; MCAT, malonyl CoA-acyl carrier protein transacylase; OXSM, 3-oxoacyl-ACP synthase; CBR4, carbonyl reductase 4; HSD17β, 3-ketoacyl-ACP reductase alpha subunit; HTD2, hydroxyacyl-thioester dehydratase type 2; MECR, mitochondrial 2-enoyl thioester reductase; LIAS, lipoic acid synthetase; Lipt1, lipoyltransferase 1; DLAT, dihydrolipoamide S-acetyltransferase; PDHX, pyruvate dehydrogenase protein X component; DLST, dihydrolipoyllysine residue succinyltransferase component of 2-oxoglutarate dehydrogenase complex; DBT, dihydrolipoyl acyltransferase.

One of the two cellular pathways responsible for fatty acid biosynthesis is localized in the mitochondria. The main pathway typical in mammals, represented by fatty acid synthase (FAS) type I, is localized in the cytosol [16], has been well characterized [43]. Mitochondrial fatty acid biosynthesis (mtFAS) has been described in yeast, where it has been reported to be involved in tRNA processing [44]. In humans, the involved enzymes have not yet all been determined, and their possible involvement in tRNA processing has not yet been demonstrated; however, some experiments have suggested the role of mammalian mtFAS. Knockdown studies in cells and mice have demonstrated that alterations in mtFAS pathway components may result in severe clinical phenotypes underlying the important physiological role of this pathway in mammalian systems [45,46,47,48]. The primary function of mtFAS is to supply octanoyl-CoA moieties for the biosynthesis of lipoic acid [16,49], an essential cofactor for several mitochondrial enzymes involved in glucose oxidation and energy metabolism (Figure 2) such as α-ketoglutarate dehydrogenase (α-KGDH), pyruvate dehydrogenase (PDH), branched-chain keto-acid dehydrogenases (BCKDH), and glycine cleavage system [38,49].

Acyl-CoA synthetase family member 3 (ACSF3) is the first enzyme of the mtFAS pathway. Defects in ACSF3 enzymes result in combined malonic and methylmalonic aciduria (CMAMMA) (OMIM: 614265) [50]. The childhood-onset form presents with symptoms such as ketoacidosis, hypoglycemia, coma, failure to thrive, and/or developmental delay, suggestive of an intermediary metabolic disorder, while the adult onset presents with neurologic manifestations including dystonia, seizures, psychiatric diseases, memory problems, and/or cognitive decline [51]. Since a very recent report unveiled the benign clinical course of CMAMMA in patients with *ACSF3* mutations, its clinical relevance has remained controversial [52]. Genetic studies in families and follow up of patients identified through newborn screen quite strongly argue against any clinical relevance to this deficiency [52]. On the other hand, the identification of adult patients with neurological problems is strongly suggestive of a chronic process leading to the late onset of symptoms. This process can be influenced by several factors such as the penetrance of mutations on the ACSF3 enzyme activity and the ability of the mammalian mitochondrial isoform of acetyl-CoA carboxylase (ACC1) to cover in part ACSF3 function and provide mitochondrial malonyl-CoA [53]. Recently, it has been reported that mutations of the enzyme in the last step of the mtFAS pathway lead to the development of the neurodegenerative disease mitochondrial enoyl-CoA reductase protein-associated neurodegeneration (MEPAN), an autosomal recessive disease resulting from mutations in the enoyl-acyl carrier protein reductase (MECR) protein of mtFAS (Heimer et al., 2016). Moreover, decreased expression of 17βHSD8 has been reported to increase susceptibility for oral squamous cell carcinoma of the head and neck [54]. Mitochondrial recessive optic atrophy disease has also been described in patients with mutations on the *MCAT* gene encoding the malonyl-CoA-acyl carrier protein transacylase [55]. Other diseases specifically caused by defects of the lipoic acid biosynthesis include mutations in lipoic acid synthase (*LIAS*) (OMIM: 614462), lipoyltransferase 1 (*LIPT1*) (OMIM: 616299) and lipoyloctanoyl transferase 2 (*LIPT2*) (OMIM: 617668) and are characterized by nonketotic hyperglycinemia, early-onset convulsions, encephalopathy, cardiomyopathy, and early death [49]. Mutations in *LIPT2* or *GCSH* are expected to cause a LIAS-like phenotype with combined involvement of GCS and the 2-oxoglutarate dehydrogenases [49]. Similarly, biallelic mutations in *LIPT2* cause a defect in the mitochondrial lipoylation which is associated with severe neonatal encephalopathy [56].

### 2.4. Metabolic Flexibility and Interconnection between Mitochondrial β-Oxidation and Fatty Acid Biosynthesis

Metabolic flexibility has been described in several diseases such as type II diabetes [57], obesity [58], cancer [59], sepsis, inflammation [60], and cardiovascular diseases [61]. One of the major organ systems in which metabolic flexibility occurs is the skeletal muscle system, with its adaptive metabolic responses during exercise [62]. Several studies on skeletal muscle have been performed to investigate the metabolic response of skeletal muscle to factors such as lipid overload and exercise. One main effect is the metabolic effect of AMP-activated protein kinase (AMPK) activation, which is able to cause a metabolic switch from fat biosynthesis to fat oxidation, promoting muscle glucose uptake [63]. Mitochondrial function has also been shown to be related to better metabolic flexibility and insulin sensitivity [64]. Changes in the oxidative phosphorylation activity protect the livers of mice from hepatic steatosis induced by a high-fat diet [65]. In addition to the skeletal muscles, adipose tissue can greatly participate in metabolic flexibility of an organism in response to exercise by reducing the size of adipocytes and improving the insulin sensitivity in both adipose and muscle tissues, which enhance adipose and skeletal glucose metabolism [62]. The heart is well known to use a concert of substrates to maintain its contractile function [17]. Physiological hypertrophy also involves a compensatory and adaptive mechanism that results in reducing wall stress and maintaining output [66]. However, in heart failure, the cardiac metabolic flexibility is clearly impaired and is associated with cardiac dysfunction and impaired energy metabolism [17,67]. Although several inherited metabolic diseases are characterized by energy deficiency, how defects affect the metabolic flexibility of cells and tissues has not yet been described. A recent report on fibroblasts from ACSF3-deficient patients showed an alteration in mitochondrial energy homeostasis [68]. Respiration studies of whole cell oxygen consumption have demonstrated reduced mitochondrial respiration in these cells accompanied by a reduction of glycolytic flux. Stable isotope labeling by amino acids in cell culture (SILAC)-based and targeted proteomic experiments have confirmed the impaired biosynthesis of octanoate (C8)-ACP and lipoic acid [68]. Because C8-ACP is essential for proper assembly of oxidative phosphorylation (OXPHOS) [69], a reduction of cellular respiratory capacity is likely due to defective mtFAS [68]. However, ACSF3-deficient cells have shown adaptive compensatory up-regulation of fatty acid β-oxidation and a strong dependency on the degradation of fatty acids for energy production, as demonstrated by Seahorse and proteomic studies [68].

As some organs and cells, to a great extent, are unable to efficiently rely on the degradation of fatty acids for energy production, we speculated that they may especially suffer from a defect in mtFAS [70]. This is the case of the very recently described disease involving the last step of mtFAS, defined as mitochondrial enoyl-CoA reductase protein-associated neurodegeneration (MEPAN) [71]. Patients present with childhood-onset dystonia, optic atrophy, and basal ganglia signal abnormalities, thereby confirm the inability of neurological cells with defects in mtFAS to compensate for a deficient energy pathway. The importance of mtFAS for mitochondrial respiratory efficiency and the regulation of mitochondrial energetics has been previously acknowledged [72,73] as well as the involvement of this pathway in the metabolic and morphologic transdifferentiation of muscle fiber types described in VLCAD^−/−^ mice [38]. Indeed, the skeletal muscle of VLCAD^−/−^ mice can compensate for defective β-oxidation by inducing an adaptive switch toward enhanced glycolysis in muscle fiber type II [37,38], accompanied by the up-regulation of mtFAS and, subsequently, the biosynthesis of lipoic acid. Especially in this regard, the role of lipoic acid in mitochondrial energy metabolism has been well established [74]. Due to its effect on PI3K and AMPK signaling and on the transcription factor PGC1α, lipoic acid stimulates glucose uptake and glycolytic flux [75], as shown in proteomic studies of white skeletal muscle in VLCAD^−/−^ mice, which have confirmed the enhanced expression of glycolytic proteins and enzymes involved in the pyruvate and carbohydrate metabolic pathways. Immunohistochemical analysis also confirmed a marked reduction in muscle oxidative fiber type I and an increase in glycolytic fiber type II [38]. These findings on ACSF3-deficient cells and VLCAD^−/−^ mice are strongly suggestive of the co-regulatory role of mtFAS and β-oxidation in energy metabolism, and a deficiency in either of these pathways can, indeed, significantly impair cellular metabolic flexibility.

### 2.5. The Effect of Diet and Sex on Metabolic Flexibility in VLCAD Deficiency

Several factors may affect energy regulatory mechanisms, including age, sex, energy requirements, and nutrient sensing induced by diet [76].

Diet has a considerable role in metabolic flexibility, depending on the type of nutrients and the period of fasting [77]. It is well known that a decrease in circulating dietary carbohydrates and lipids and a decline in insulin/glucagon ratio during fasting induce a switch toward fatty acid oxidation [77]. In line with these studies, other reports have shown that subjects under a high-fat diet were able to increase fatty acid oxidation at the expense of the glycolytic rate, though this effect was not observed in obese individuals. Very likely these individuals are unable to up-regulate PDK4, which inhibits the pyruvate dehydrogenase complex, and they therefore did not show a fast response to the enhancement of circulating lipids [78]. Skeletal muscle is particular sensitive to nutrient availability; the stimulation of insulin production increases glucose transport into the cell and the uptake of triglycerides from the blood into muscle, and at the same time, decreases the rate of fatty acid oxidation and increases the rate of protein synthesis [79]. In the white skeletal muscle of VLCAD^−/−^ mice, adaptive compensatory up-regulation mediated by mtFAS, toward glycolysis in type II muscle fibers, was reversed by prolonged supplementation with an MCT diet [38], likely in response to nutrient availability. In fact, MCTs are rapidly hydrolyzed after ingestion, and the circulating medium-chain fatty acids can be easily uptaken up by peripheral tissue and undergo β-oxidation as they can cross the mitochondrial membrane without the need for an active transport system [80].

The involvement of mFAS in up-regulating glucose oxidation has not been previously described and underlines its relevance in the maintenance of metabolic flexibility. In a similar manner, sex represents an important variable due to the significant influence of hormonal changes and even lifestyle [76]. In the literature, there are several reports that describe sex-specific differences in basal metabolism; however, the data are very controversial [81,82,83]. Basal metabolism represents the minimum energetic rate required by an organism at rest [84]. On the one hand, some reports have shown higher efficiency in mitochondria isolated from the heart, skeletal muscles, and liver of male B6(C57Bl/6J) mouse strain [82]. On the other hand, it has been shown that mitochondria isolated from the cardiac muscle of female rats are fewer in number but morphologically more differentiated than those of male rats, indicative of a more efficient mitochondrial electron transport chain and lower H_2_O_2_ generation [83]. Similarly, mitochondria from tissues other than heart have also displayed higher energetic efficiency and mitochondrial oxygen consumption [81,85]. These studies are in line with the higher respiration rate observed in fibroblasts from wildtype (WT) female mice, reported by Seahorse experiments [42]. However, when cells were incubated with octanoate (C8), one of the major components of MCTs, the nutrient availability changed and mitochondrial respiration was more efficiently enhanced in WT male mice, suggesting that an overload of available substrate may, in part, hamper the process involved in the metabolic flexibility in female cells or that females have a higher mitochondrial oxygen consumption rate when using non-fatty acid substrates [42]. These findings are in accordance with previous observations on the development of severe metabolic syndrome in female VLCAD^−/−^ mice on a long-term MCT diet, whereas male mice were protected [35]. Moreover, incubation with C8 has also shown a completely different response and sex-specific activation of signaling pathways, in particular, sex-specific activation of mTORc1 was observed in female mice. The central role of mTOR signaling in regulating both lipogenesis and lipolysis has been acknowledged for a long time [86]. In this regard, incubation of female VLCAD^−/−^ mouse fibroblasts with C8 has led to the activation of the ERK/mTORc1 pathway and, subsequently, of lipogenesis [42], corroborating the previously reported sex-specific development of a metabolic syndrome in female VLCAD^−/−^ mice after prolonged MCT supplementation [35]. These findings were also confirmed by gene expression analysis and SILAC-based proteomic analysis in fibroblasts, which showed clear, subsequent stimulation of de novo fatty acid biosynthesis. Although lipogenesis was also up-regulated in cells of male mice, mTORc1 was not activated by C8. Under control conditions, cells from male VLCAD^−/−^ mice already show up-regulated levels of ERK1/ERK2 factors; C8 had no further effects. In addition, it has been shown that medium-chain fatty acids are able to occupy the ligand binding pocket (LBP) of the ligand binding domain (LBD) of the peroxisome proliferator-activated receptor (PPAR)γ and are therefore considered to be selective PPARγ activators [87]. The data are in line with the up-regulation of PPARγ after incubation with C8, with the subsequent increment in peroxisomal activity reflected by higher peroxisomal β-oxidation and biogenesis [42]. These observations clearly support the fact that sex and diet play a key role in the regulation of energy homeostasis and metabolic flexibility in fatty acid oxidation disorders, and that these two variables should likely be taken into consideration in the long-term treatment of FAOD patients.

### 2.6. The Biological Role of Mitochondrial Fatty Acid Biosynthesis (mtFAS) in Energy Regulation

The localization of the mtFAS pathway in mitochondria is thought to be due to an ancient endosymbiotic event in which mitochondria were generated from bacteria [88]; mtFAS is responsible for the mitochondrial biosynthesis of fatty acids of chain length up to C8, which are used as precursors for the biosynthesis of lipoic acid. Each protein in this pathway catalyzes a separate step, in contrast with the microbial fatty acid synthase type I (FASI), which consists of a multifunctional complex and is responsible for the synthesis of fatty acids of chain length up to C16/C18. From this point of view, mtFAS’s practical function has long been recognized [89]. However, the recognition of its role in the regulation of metabolic flexibility and energy homeostasis is rather new and clear details have only recently emerged [68,72]. In yeast, mtFAS it is known to be involved in mitochondrial respiration, tRNA processing, and control of mitochondrial gene expression [16]. Very recently, it has been suggested that the biosynthesis of malonyl-CoA catalyzed by ACSF3 is important for regulation of mitochondrial pathways as this compound is used for the post-translational malonylation of lysine residues in mitochondrial proteins. This process markedly improves mitochondrial efficiency [90] and makes the role of ACSF3 in the regulation of energy metabolism even more relevant. Post-translational modifications of mitochondrial proteins have already been described in the regulation of energy homeostasis. The most common process is the protein acetylation. The process of acetylation and deacetylation of mitochondrial proteins modulated by SIRT3 plays a role in cardiac dysfunction [91]. This protein also deacetylates and enzymatically activates the fatty acid oxidation enzyme long-chain acetyl-CoA dehydrogenase (LCAD) [92]. The importance of the mtFAS pathway emerged very clearly in our studies on the metabolic and morphologic switch in muscle fibers of VLCAD^−/−^ mice, which occurs via up-regulation of mtFAS and lipoic acid biosynthesis [38]. Lipoic acid is found in all kingdoms of life and is an essential cofactor for several cellular redox reactions [49]. Several inborn errors of metabolism also characterized by defective lipoylation have been described [49], such as lipoic acid synthetase (LIAS) [93,94] and lipoyltransferase 1 (LIPT1) deficiencies [95]. As in most of the identified patients the onset of clinical manifestations appeared later in life [50], and pre-symptomatic patient management may have contributed to reduce the initial risk of symptoms development [52], we hypothesize that compensatory mechanisms in mitochondrial energetics may specifically address the required energy need. Mutations in the ACSF3 gene may also result in lipoylation impairments [68] and dysregulated OXPHOS assembly, as also recently shown in mtFAS mutant mouse skeletal fibroblast cell lines [96]. Similarly, ACFS3-deficient myoblasts displayed severe impairment in the composition of complex lipids [68]. Of particular interest was the finding that an increased concentration of cardiolipins was associated with an accumulation of species with longer fatty acid chain lengths and higher desaturation degrees. Abnormal cardiolipin profile is also typical for the severe and rare mitochondriopathy called Barth syndrome [97], presenting with cardiomyopathy (CM), skeletal myopathy, growth delay, neutropenia, and increased urinary excretion of 3-methylglutaconic acid (3-MGCA). These data would strongly suggest that mtFAS may not only be involved in the regulation of energy metabolism but also in lipid modeling and homeostasis [68].

## 3. Conclusions

Inherited metabolic defects of mitochondrial fatty acid metabolism affect the ability of cells to maintain bioenergetic balance. On the one hand, VLCAD^−/−^ mice undergo adaptational changes characterized by metabolic and morphologic up-regulation of glycolytic muscle fiber type II. The up-regulation of glucose oxidative metabolism, observed in VLCAD^−/−^ mouse model, occur in parallel with the up-regulation of the mitochondrial fatty acid synthase mtFAS. Dietary interventions in the form of medium-chain fatty acids differently activate signaling pathways and result in the sex-specific development of metabolic syndrome in VLCAD^−/−^ mice. On the other hand, fibroblasts from patients with homozygous mutations in the ACSF3 gene display reduced glycolytic flux and mitochondrial respiration followed by adaptive dependency on β-oxidation of fatty acids for energy supply.

## Data Availability

Not applicable.
